# Protein Detection Using the Multiplexed Proximity Extension Assay (PEA) from Plasma and Vaginal Fluid Applied to the Indicating FTA Elute Micro Card™

**DOI:** 10.5772/64000

**Published:** 2016-01-01

**Authors:** Malin Berggrund, Daniel Ekman, Inger Gustavsson, Karin Sundfeldt, Matts Olovsson, Stefan Enroth, Ulf Gyllensten

**Affiliations:** 1 Department of Immunology, Genetics and Pathology, Science for Life Laboratory, Biomedical Center, Uppsala University, Uppsala, Sweden; 2 Olink Bioscience AB, Dag Hammarskjölds väg, Uppsala, Sweden; 3 Department of Obstetrics and Gynecology, Institute for Clinical Sciences, Sahlgrenska Cancer Center, Gothenburg University, Gothenburg, Sweden; 4 Department of Women's and Children's Health, Uppsala University, Uppsala, Sweden

**Keywords:** FTA Elute Card, Vaginal Fluid, Plasma, Proximity Extension Assay

## Abstract

The indicating FTA elute micro card™ has been developed to collect and stabilize the nucleic acid in biological samples and is widely used in human and veterinary medicine and other disciplines. This card is not recommended for protein analyses, since surface treatment may denature proteins. We studied the ability to analyse proteins in human plasma and vaginal fluid as applied to the indicating FTA elute micro card™ using the sensitive proximity extension assay (PEA). Among 92 proteins in the Proseek Multiplex Oncology Iv2 panel, 87 were above the limit of detection (LOD) in liquid plasma and 56 among 92 above LOD in plasma applied to FTA cards. Washing and protein elution protocols were compared to identify an optimal method. Liquid-based cytology samples showed a lower number of proteins above LOD than FTA cards with vaginal fluid samples applied. Our results demonstrate that samples applied to the indicating FTA elute micro card™ are amendable to protein analyses, given that a sensitive protein detection assay is used. The results imply that biological samples applied to FTA cards can be used for DNA, RNA and protein detection.

## 1. Introduction

A number of sampling devices have been developed to collect blood or plasma/serum for protein analyses, ranging from solid phase matrices to the recent use of nanocapillary technology [1]. The collection of blood spots, buccal cells and saliva on filter paper is a common sampling method, used for the screening of newborns for inherited diseases, in forensic medicine and for many other purposes. Specific surface treatments have been developed to stabilize nucleic acids applied onto filter paper. Whatman FTA technology consists of two distinct chemistries, both of which have the ability to lyse cells on contact, denature proteins and protect DNA and RNA from degradation. The FTA elute card™ (GE Healthcare, United Kingdom) contains a chaotropic salt and proteins remain tightly bound while DNA is eluted from the matrix. The indicating FTA elute micro card™ also includes a purple dye that turns white when a clear sample is applied. This colour change is useful for verifying that the biological sample has been deposited on the card surface, specifically when the card is used for self-collection of samples. The indicating FTA elute micro card™ allows for a simple elution of DNA and RNA from the surface of the card, and is extremely suitable for genotyping, sequencing and real-time PCR applications [2, 3].

The indicating FTA elute micro card™ is presently used for the self-collection of cervico-vaginal fluid (CVF) samples in cervical cancer screening programmes [4–10]. It will therefore be of interest to determine if samples collected onto the FTA card™ can also be used for analyses of protein biomarkers. The FTA elute card is not recommended for protein analyses, since surface treatment may denature proteins and to the best of our knowledge, no study has been performed to determine whether proteins applied to the FTA elute micro card™ can be detected. The aim of this study was to examine the potential for using the indicating FTA elute micro card™ for protein analyses by employing the very sensitive and highly specific protein extension assay (PEA) [11, 12]. PEA is based on the use of two antibodies towards the target protein, providing high specificity. Each antibody has a DNA tail attached and if the DNA tails on the two antibodies are within reach of each other, they can act as a substrate for DNA polymerase. Protein detection is achieved through a real-time PCR, resulting in high analytical sensitivity. The PEA has previously been used to measure protein in biomarker studies [13, 14]. If protein detection is indicated as feasible in samples applied to the indicating FTA elute micro card™, it will offer a quick, cost-effective, easy-to-store option for sample collection, both for diagnostic purposes and population screening.

## 2. Samples and Methods

### 2.1 Samples and preparation

CVF samples were collected from women participating in the organized cervical cancer screening using the Rover Viba brush (Rover Medical Devices B.V., Oss, The Netherlands) and applied to the indicating FTA elute micro card™ as described earlier [15].

All FTA card samples were selected from HPV-negative women. One sample, which had been stored for two weeks, was selected for a first buffer-test. Twenty-eight CVF samples were gathered representing a storage time on the card of 1–29 months prior to analysis. Twelve samples had been stored for one month, six samples for seven months, six samples for 19 months and four samples were stored for 29 months. Five additional samples, with storage times of less than two weeks, were selected for the testing protocols for washing.

Plasma samples were obtained from five women. For each woman, 10 μl plasma aliquots were applied to an indicating FTA elute micro card™; the card surfaces were dried and stored at room temperature for either 24 h or 72 h. All samples applied to the FTA cards were processed using the 3 mm Ø Harris micro punch (Whatman, Inc., Clifton, NJ) for collecting punches as previously described [15]. The following washing procedures were compared: I) no wash prior to elution; II) 1 × 5 min. wash in 100 μl water; III) 2 × 5 min. wash in 100 μl water. Proteins were eluted from the indicating FTA elute micro card™ by placing the punch in either a 20 μl Radioimmunoprecipitation assay buffer (RIPA) or 20 μl Phosphate-buffered saline (PBS)-Tween 20 (0.05 %) buffer for 60 to 90 minutes.

We also investigated samples from 28 women that were collected using the procedure applied in liquid-based cytology. These samples were collected using a cytobrush and dissolved in a 20 ml ThinPrep storage solution (Hologic, Inc. San Diego, USA), and stored at room temperature for 3–10 days prior to freezing. From this solution, 1 μl was used for the PEA analysis.

### 2.2 Protein analysis

One μl of either liquid plasma, elution from FTA card, or liquid-based cytology sample was analysed using the proximity extension assay (PEA) and the Proseek Multiplex Oncology I v2 panel (Olink Bioscience AB, Uppsala, Sweden). This panel includes 92 proteins selected on the basis of their reported associations with cancer. The protein analysis is reported as normalized protein expression levels (NPX), which are Ct values normalized by the subtraction of values for extension control, as well as an interplate control; the scale is shifted using a correction factor (normal background noise) and reported in Log 2 scale. All assay characteristics including detection limits and measurements of assay performance and validations are available from the manufacturer's webpage (http://www.olink.com/products/proseek-multiplex/downloads/).

### 2.3 Statistical analysis

All analyses and illustrations were performed in R [16]. Spearman correlations were used throughout. The significance of differences in distributions was calculated using the double-sided Wilcoxon signed-rank test. Resulting *P*-values were corrected for multiple testing using either Bonferroni or FDR correction and considered significant if the q-value < 0.05. Linear modelling for estimating the correlation between the NPX measurement and storage time was performed with the lm function and the ANOVA function.

## 3. Results

### 3.1 Elution buffer optimization

**Figure 1.**
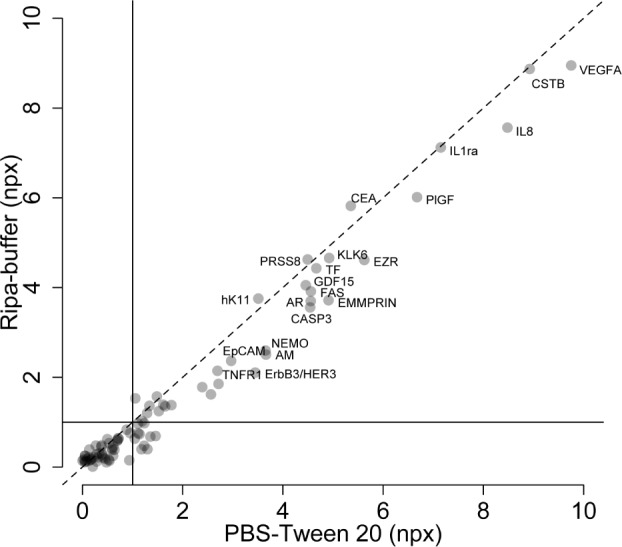
Comparison of the NPX values for the 92 proteins in the Proseek Multiplex Oncology Iv2 panel from analysis of a sample applied to the indicating FTA elute micro card™ and eluted with either RIPA (y-axis) or PBS-Tween 20 buffer (x-axis). PBS-Tween reports slightly higher NPX-values for the majority of the 92 proteins.

We first compared two elution buffers, PBS-Tween 20 (0.05 %) and RIPA, for their efficiency in terms of recovering proteins from the indicating FTA elute micro card™. A single punch from one FTA sample was eluted in 20 μl PBS-Tween 20 (0.05 %) or RIPA buffer and analysed using the Proseek Multiplex Oncology I v2 panel. For both buffers, 80 of the 92 proteins in the panel had levels above the assay background (Spearman's rho, R^2^=0.92, p-value < 2.2e-16) (Figure 1). With a cut-off of >1 NPX above background, the PBS-Tween 20 buffer resulted in the detection of 43 proteins and the RIPA buffer in 33 proteins. Additionally, 60 proteins showed higher NPX values for the sample FTA elution card than a blank FTA elution card for PBS-Tween 20 buffer and 52 proteins for the sample eluted in RIPA buffer. Thus, the PBS-Tween 20 buffer performed better than the RIPA buffer and was therefore used in the following analyses.

### 3.2 Proteins are measurable from the FTA cards

We next compared liquid plasma samples with plasma applied to the indicating FTA elute micro card™ and stored for either 24 h or 72 h. One punch from each FTA card was eluted in 20 μl PBS-Tween 20 buffer and analysed using the Proseek Multiplex Oncology I v2 panel. As expected from the elution protocol, the plasma sampled and applied to the FTA showed generally lower protein amounts than the liquid plasma samples. In total, 87 of the 92 proteins in the panel were detected in all liquid plasma samples and 56 and 52 proteins were detected in the samples on FTA cards stored for 24 h and 72 h, respectively (Table 1).

**Table 1.** List of proteins and percentage of samples over limit of detection (LOD) for each protein when using liquid plasma (left column) plasma on indicating FTA elute micro card™ stored for 24h (middle column) and 72h (right column)

Protein	Plasma (% of samples over LOD)	24 h (% of samples over LOD)	72 h (% of samples over LOD)
IL-8	100%	100%	100%
VEGF-A	100%	100%	100%
AM	100%	100%	100%
GDF-15	100%	100%	100%
PlGF	100%	100%	100%
CSF-1	100%	100%	100%
CSTB	100%	100%	100%
MCP-1	100%	100%	100%
KLK6	100%	100%	100%
TRAIL-R2	100%	100%	100%
LAP-TGF-beta-1	100%	100%	100%
TF	100%	100%	100%
TNF-R1	100%	100%	100%
PDGF-subunit-B	100%	100%	100%
PARK7	100%	100%	100%
CXCL11	100%	100%	100%
VE-statin	100%	100%	100%
IL-7	100%	100%	100%
SCF	100%	100%	100%
CXCL9	100%	100%	100%
TNF-R2	100%	100%	100%
TNFSF14	100%	100%	100%
GH	100%	100%	100%
FasL	100%	100%	100%
FAS	100%	100%	100%
CCL19	100%	100%	100%
EMMPRIN	100%	100%	100%
CXCL10	100%	100%	100%
Ep-CAM	100%	100%	100%
HGF	100%	100%	100%
LITAF	100%	100%	100%
CXCL5	100%	100%	100%
MK	100%	100%	100%
U-PAR	100%	100%	100%
CDH3	100%	100%	100%
LYN	100%	100%	100%
Flt3L	100%	100%	100%
HB-EGF	100%	100%	100%
CD69	100%	100%	100%
TR-AP	100%	100%	100%
CDKN1A	100%	100%	100%
REG-4	100%	100%	100%
VEGF-D	100%	100%	100%
HE4	100%	100%	100%
CXCL13	100%	100%	100%
CA-125	100%	100%	100%
PRSS8	100%	100%	100%
PECAM-1	100%	100%	100%
FR-alpha	100%	100%	100%
TIE2	100%	100%	80%
MIC-A	100%	100%	80%
IL-6	100%	100%	60%
ICOSLG	100%	100%	60%
FS	100%	100%	60%
NTRK3	100%	100%	20%
ILT-3	100%	80%	80%
VEGFR-2	100%	80%	80%
ITGA1	100%	80%	80%
ErbB3-HER3	100%	60%	80%
MIA	100%	60%	80%
PTPN22	100%	60%	60%
SELE	100%	60%	40%
FUR	100%	60%	40%
AR	100%	60%	20%
eIF-4B	100%	40%	40%
EZR	100%	40%	20%
PRL	100%	40%	20%
IL-12	100%	20%	40%
MMP-1	100%	20%	40%
ErbB2-HER2	100%	20%	40%
TNFRSF4	100%	20%	40%
IL-6RA	100%	20%	20%
BAFF	100%	20%	20%
ErbB4-HER4	100%	20%	20%
NEMO	100%	20%	20%
FADD	100%	20%	0%
IFN-gamma	100%	20%	0%
VIM	100%	0%	40%
CASP-3	100%	0%	20%
CD40-L	100%	0%	0%
IL-1ra	100%	0%	0%
hK11	100%	0%	0%
CAIX	100%	0%	0%
TGF-alpha	100%	0%	0%
EGFR	100%	0%	0%
THPO	100%	0%	0%
IL-17RB	100%	0%	0%
CEA	80%	20%	20%
MYD88	80%	0%	20%
EPO	80%	0%	0%
IL-2	60%	80%	100%
TNF	40%	100%	100%

**Figure 2.**
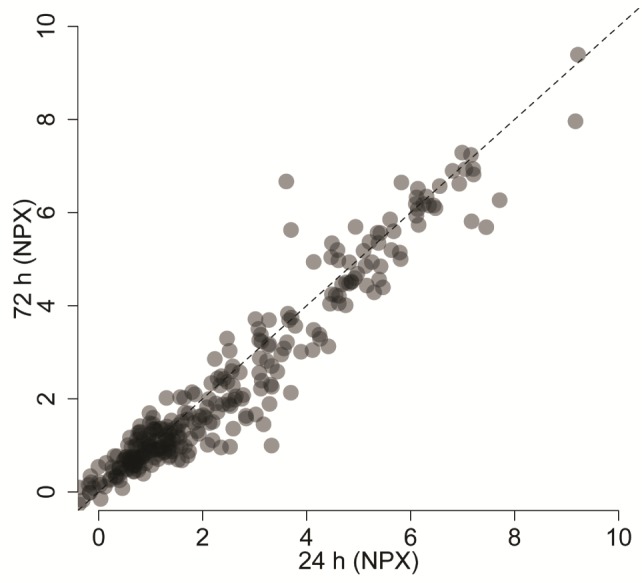
Correlation between NPX values for all observations with measurements above the limit of detection at two time-points from cervicovaginal fluid samples stored on FTA cards for 24 h (x-axis) and 72 h (y-axis), Spearman's rho, R^2^=0.89, p-value < 2.2e-16

The correlation between all observations for all proteins between 24 h and 72 h was high (Spearman's rho, R^2^=0.89, p-value < 2.2e-16) (Figure 2). In a per-protein analysis, replacing values below the limit of detection with protein specific LOD-values, five proteins (CSF-1, NTRK3, ICOSLG, SCF and PECAM-1) were found to have nominally significant differences between the two time-points (p < 0.05, Wilcox test). None of these associations remained significant after multiple hypothesis corrections. Only two proteins showed a mean difference of more than 1 NPX unit between samples stored for 24 h and 72 h (CSF-1 and TNF-R1, both of which had lower NPX values at 72 h).

### 3.3 Washing optimization

**Figure 3.**
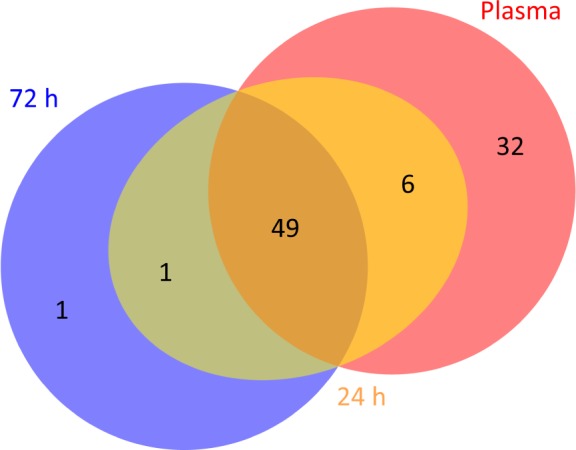
The overlap in proteins above LOD in 100% of the samples when analysing liquid plasma (plasma), plasma samples applied to the indicating FTA elute micro card™ and stored for 24 h (t24) and 72 h (t72) prior to analysis

There was a large overlap between the proteins that were detectable in liquid plasma and on the FTA cards. Only six proteins were detectable in liquid plasma and on FTA cards stored for 24 h, but not on FTA cards stored for 72 h (Figure 3). Twelve out of 15 plasma samples on the FTA cards did not pass the Proseek internal controls for incubation, extension and detection. Nonetheless, 56 of 92 proteins were detected over LOD in all samples for FTA cards stored 24 h and 51 of 92 proteins were detected in all samples for FTA cards stored for 72 h. We reasoned that components in the surface chemistry of the FTA elute cards™ had a negative effect on the assay.

In order to reduce the problem regarding failed internal controls, we tested three different protocols for washing punches prior to protein elution. One punch from each of 28 indicating FTA elute micro cards™ with a vaginal fluid sample was analysed using one of three different protocols: I) no wash prior to elution; II) 1 × 5 min. wash in 100 μl water; III) 2 × 5 min. wash in 100 μl water. Samples were then eluted in 20 μl PBS-Tween 20 buffer and analysed using the Proseek Multiplex Oncology I v2 panel.

**Figure 4.**
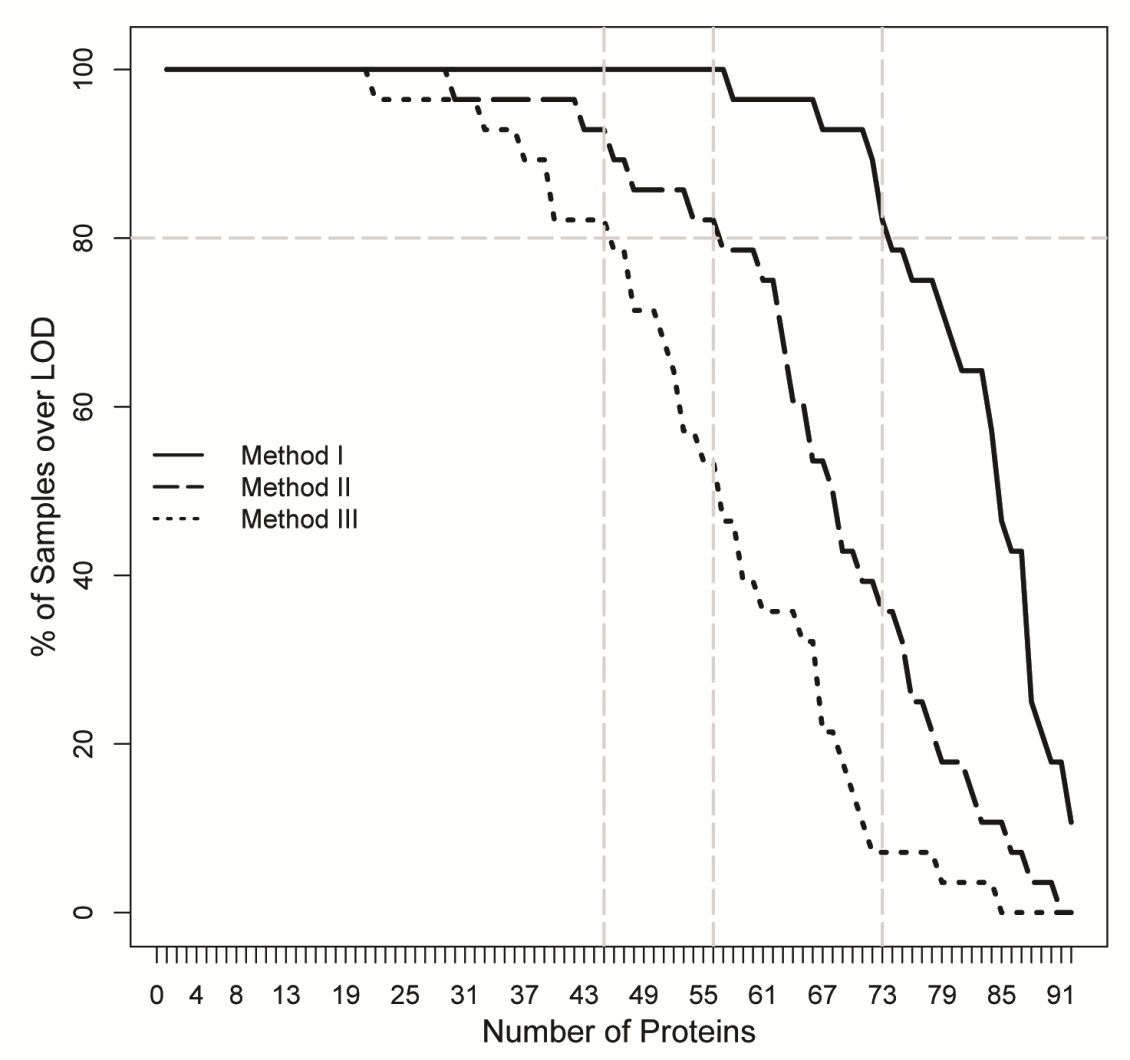
The number of proteins detected over limit of detection (LOD) for the three washing methods as a function of the percentage of samples studied with a NPX value over the LOD

With method I (no wash), eight samples showed signs of inhibition of internal controls, compared to one sample with method II and zero samples with method III. In total, 57 of 92 proteins had levels over LOD in all 28 samples using method I and 73 proteins were detected in 80% of the samples. With method II, 29 of 92 proteins were detectable in all 28 samples and 56 proteins were detected in 80% of the samples. With method III, 21 of 92 proteins had levels over LOD in all 28 samples and 45 proteins were detected in 80% of the samples. The number of proteins detected using the different extraction protocols as a function of the fraction of individuals with protein levels over LOD is shown in Figure 4.

### 3.4 Detectable proteins remain stable on the FTA-cards

The 28 samples were stored for up to 29 months. No significant effect of storage time on protein amounts was observed using Bonferroni correction (p > 0.065). With FDR correction, 19 proteins showed an effect due to storage time (p < 0.05). The urokinase plasminogen activator surface receptor (UPAR) protein had the largest difference between samples stored for one month and samples stored for 29 months, with a mean difference of 3.59 NPX (Supplementary Table S1).

### 3.5 Adding more material does not improve detectability

The additional wash improved the analysis, but may also have resulted in loss of protein. We therefore investigated whether increasing the input material, i.e., the number of punches from a sample, would result in higher protein amounts. To this end one, two, three or four punches were obtained from an FTA card with CVF sample from five women and washed for 2 × 5 min. in 100 μl water (method III), eluted in a 20 μl PBS-Tween 20 buffer and analysed using the Proseek Multiplex Oncology I v2 panel. The samples using one punch passed all the assay controls, while the samples using two punches or more did not pass the incubation control. Extension and detection controls were not affected, regardless of the number of punches used.

Independent of the number of punches used, roughly 50% of proteins in the panel showed levels above the LOD in 100% of the women (47/92 with one punch, 47/92 with two punches, 46/92 with three punches and 46/92 with four punches). When considering proteins that were measurable in more than 50% of individuals, 62 and 58 proteins were above LOD, using one and four punches, respectively. Thus, increasing the number of punches did not increase the number of proteins to a level above the LOD when using the presently used wash and elusion protocol.

### 3.6 Comparison with liquid cytology samples

For comparison we also investigated samples collected with the procedure used in liquid-based cytology (Thin-Prep transport solution). A total of 28 cytology samples were analysed using the Proseek Multiplex Oncology I v2 panel. Two of the samples did not pass the internal assay controls and were removed from the analysis. Only seven proteins were above the LOD in 100% of the 26 cytology samples, compared to 21 proteins that were above the LOD in 100% of the FTA samples (method III) (Table 2).

**Table 2.** List of proteins and percentage of samples over the limit of detection (LOD) among the indicating FTA elute micro card™ samples (left column) and the Liguid-based cytology samples (right column); 2 of the 28 Liguid-based cytology samples did not pass the quality controls and were excluded. 7 proteins were detected in all 26 Liguid-based cytology samples, compared to 21 proteins that were detected in the 28 FTA samples.

Protein	FTA elute micro cardTM (% of samples over LOD)	Protein	Liquid cytology samples (% of samples over LOD)
IL-8	100%	IL-8	100%
VEGF-A	100%	VEGF-A	100%
AM	100%	IL-1ra	100%
PlGF	100%	CSTB	100%
EZR	100%	U-PAR	100%
IL-1ra	100%	HE4	100%
CSTB	100%	PRSS8	100%
MCP-1	100%	MCP-1	96%
TNF-R1	100%	TNF-R1	96%
PARK7	100%	CEA	96%
FADD	100%	EZR	92%
FAS	100%	hK11	92%
EMMPRIN	100%	Ep-CAM	92%
LITAF	100%	CXCL5	92%
CASP-3	100%	TF	88%
CDKN1A	100%	FAS	88%
TGF-alpha	100%	EMMPRIN	88%
HE4	100%	CASP-3	88%
eIF-4B	100%	FR-alpha	88%
CA-125	100%	GDF-15	85%
PRSS8	100%	MK	85%
GDF-15	96%	TGF-alpha	85%
TF	96%	CXCL13	85%
CXCL9	96%	VIM	85%
PTPN22	96%	PlGF	81%
CXCL10	96%	IL-6	81%
CXCL5	96%	CXCL10	77%
MK	96%	HGF	77%
U-PAR	96%	AR	77%
LYN	96%	CXCL9	73%
CXCL13	96%	ErbB3-HER3	73%
CEA	96%	TNF-R2	65%
FUR	93%	TR-AP	65%
Ep-CAM	93%	KLK6	62%
AR	93%	TRAIL-R2	62%
NEMO	93%	FADD	62%
KLK6	89%	LITAF	62%
hK11	89%	NEMO	62%
ErbB3-HER3	89%	eIF-4B	62%
IL-6	82%	CA-125	58%
LAP-TGF-beta-1	82%	LAP-TGF-beta-1	50%
FasL	82%	CSF-1	46%
CD69	82%	PARK7	46%
VIM	82%	FUR	46%
FR-alpha	82%	CAIX	42%
HGF	79%	ErbB4-HER4	42%
ICOSLG	79%	CD69	31%
CSF-1	71%	MIC-A	31%
ErbB2-HER2	71%	AM	27%
MIC-A	71%	ErbB2-HER2	27%
VE-statin	68%	Flt3L	27%
CDH3	64%	CCL19	23%
Flt3L	57%	PTPN22	23%
PECAM-1	57%	LYN	23%
CCL19	54%	PECAM-1	23%
MYD88	54%	SCF	15%
ILT-3	46%	HB-EGF	15%
TNF-R2	46%	CXCL11	12%
PDGF-subunit-B	39%	BAFF	12%
TNF	39%	TNFRSF4	12%
IL-7	36%	IL-12	8%
TNFSF14	36%	FasL	8%
ErbB4-HER4	36%	MYD88	8%
ITGA1	36%	MIA	8%
TRAIL-R2	32%	CDKN1A	8%
CXCL11	32%	EGFR	8%
SCF	21%	ITGA1	8%
FS	21%	FS	8%
REG-4	18%	ILT-3	4%
HB-EGF	14%	TIE2	4%
MMP-1	11%	PDGF-subunit-B	4%
TIE2	7%	IL-2	4%
BAFF	7%	IL-7	4%
MIA	7%	IL-6RA	4%
TNFRSF4	7%	MMP-1	4%
TR-AP	7%	TNFSF14	4%
THPO	7%	GH	4%
NTRK3	7%	CDH3	4%
SELE	4%	REG-4	4%
IL-12	4%	VEGF-D	4%
PRL	4%	IL-17RB	4%
GH	4%	CD40-L	0%
CAIX	4%	SELE	0%
EGFR	4%	EPO	0%
CD40-L	0%	VE-statin	0%
EPO	0%	PRL	0%
IL-2	0%	TNF	0%
IL-6RA	0%	ICOSLG	0%
VEGFR-2	0%	VEGFR-2	0%
IFN-gamma	0%	IFN-gamma	0%
VEGF-D	0%	THPO	0%
IL-17RB	0%	NTRK3	0%

## 4. Discussion

This study shows that plasma and cervico-vaginal fluid samples applied to the indicating FTA elute micro card™ are amendable to protein analysis. The PBS-Tween (0.05 %) elution is the preferable elution buffer. The majority of proteins in the Proseek Multiplex Oncology I v2 panel that were detectable in liquid plasma were also above LOD in samples applied to the indicating FTA elute micro card™. The NPX values were generally lower in the FTA card samples, although some proteins showed high NPX values. This was to some extent expected because of the elution process, since for FTA card samples, the 10 μl of plasma in a punch was eluted in a 20 μl of buffer, resulting in half of the concentration of proteins entering the assay, compared to the liquid plasma samples.

Our comparison of washing methods showed that although extensive washing may results in a loss of protein, adding a second wash to the punches from FTA cards reduced the number of samples that did not pass the internal controls. The results showed that the proteins on the FTA card appear stable. The protein profile in the vaginal fluid samples on FTA cards from 28 women stored for up to two years showed an effect on 19 proteins, with FDR correction (p < 0.05); however, this effect was not seen using Bonferroni correction (p > 0.065). We also studied whether the number of proteins above LOD detected in the FTA card samples could be increased by adding more starting material, i.e., more punches in the washing and elution steps. However, this did not result in additional proteins appearing above LOD, but only increased the number of samples failing internal control. An alternative would be to use a protocol that selectively removes inhibitors from the FTA cards' surface prior to the elution of the proteins. The liquid cytology samples showed a slightly higher abundance of some proteins compared to samples from the indicating FTA elute micro card™, but the number of proteins with levels above LOD was higher for the FTA cards. The lower number of detectable proteins in the liquid cytology samples may have been due to the procedure for sample collection and storage, and in particular, the substantial dilution of the original sample in the transport solution.

Our study demonstrates the feasibility of measuring proteins from vaginal fluid and plasma collected onto the indicating FTA elute micro card™. This opens up the possibility of studying both DNA, RNA and protein biomarkers in a single sample applied to the indicating FTA elute micro card™. The indicating FTA elute micro card™ is extremely suitable where biobanking is concerned, since these cards can be stored at room temperature and do not require large storage space or the subzero conditions needed for liquid cytology samples.

## 5. Compliance with Ethical Research Standards

Daniel Ekman is employed at Olink Bioscience AB, Uppsala. All other authors declare no conflicts of interest.
